# Integrated Multiomics Reveals Gut–Brain Axis Dysregulation and Phenotype-Specific Metabolic Signatures in Children with Febrile Seizures

**DOI:** 10.3390/biomedicines14071568

**Published:** 2026-07-13

**Authors:** Xin Zhang, Lingyan Ma, Yang Wen, Feng Gao, Yingping Xiao, Jianhua Mao

**Affiliations:** 1 Children’s Hospital, Zhejiang University School of Medicine, National Clinical Research Center for Children and Adolescents Health and Diseases, Hangzhou 310052, China; 6510039@zju.edu.cn (X.Z.); epilepsy@zju.edu.cn (F.G.); 2 State Key Laboratory for Quality and Safety of Agro-Products, Institute of Agro-Product Safety and Nutrition, Zhejiang Academy of Agricultural Sciences, Hangzhou 310021, China; maly1124@163.com (L.M.); weny@zaas.ac.cn (Y.W.)

**Keywords:** febrile seizures, gut microbiota, amino acid metabolism, metabolomics, arginine biosynthesis

## Abstract

**Background:** Febrile seizures (FSs) are the most common neurological emergency in early childhood; however, the biological basis of disease heterogeneity remains poorly understood. Although growing evidence suggests that gut–brain axis dysregulation contributes to seizure susceptibility, it remains unclear whether gut microbiota-associated metabolic disturbances are linked to clinical phenotypes, particularly simple FS (SFS) and complex FS (CFS). **Methods:** An integrated multiomics study was conducted in clinically characterized pediatric cohorts, comprising 50 children with FS and 50 healthy controls, and their gut microbiota was profiled via 16S rRNA sequencing. As some pediatric serum specimens did not meet the minimum volume requirement of the analytical platform, serum amino acid profiling was performed in a subset of samples using an equal-volume pooling strategy. In brief, two individual serum samples from the same study group were combined into one composite sample, yielding 25 pooled samples in the FS group and 25 in the control group. Subsequently, untargeted fecal metabolomics was performed in an expanded cohort of 53 healthy controls, 50 children with SFS, and 42 children with CFS. Additionally, the central metabolic profiles of the CFS and SFS groups were compared using untargeted cerebrospinal fluid metabolomics. Given the variation in sample sizes across omics platforms, each dataset was analyzed within its corresponding eligible subset, and cross-omics integration was interpreted primarily at the pathway and phenotype levels. **Results:** Children with FS exhibited reduced gut microbial diversity and altered microbial composition, characterized by the enrichment of *Streptococcus*, *Enterococcus*, and *Escherichia–Shigella*, along with the depletion of beneficial taxa, including *Faecalibacterium*, *Lachnoclostridium*, and *Parasutterella*. Functional prediction indicated significant changes in amino acid-related pathways, especially arginine and proline metabolism, amino acid metabolism, and glutathione metabolism. Serum profiling showed elevated levels of phenylalanine, kynurenine, and γ-aminobutyric acid, along with reduced levels of tryptophan, threonine, lysine, glutamine, taurine, citrulline, 3-methylhistidine, α-aminobutyric acid, hydroxyproline, and phosphoethanolamine. Correlation analysis identified *Lachnoclostridium* and *Parasutterella* as key taxa associated with neuroactive metabolites. Additionally, fecal metabolomics revealed that both SFS and CFS samples exhibited significant metabolic divergence from the controls, with arginine biosynthesis emerging as a shared altered pathway and L-arginine reduced in both phenotypes. Notably, cerebrospinal fluid metabolomics demonstrated clear metabolic separation between CFS and SFS, signifying phenotype-specific central metabolic signatures. **Conclusions:** FS is related to gut microbiota dysbiosis, systemic amino acid remodeling, and phenotype-associated metabolic stratification. Arginine metabolism may represent a shared mechanistic hub across FS phenotypes, while central metabolic divergence may contribute to the biological distinction between SFS and CFS. These findings establish a multiomics framework for understanding FS pathogenesis and identifying potential biomarkers and therapeutic targets.

## 1. Introduction

Febrile seizures (FSs) represent the most common seizure-related neurological events in early childhood, affecting approximately 2–5% of children younger than 5 years old [1,2]. Most FSs are self-limited and benign; however, prolonged, focal, or recurrent episodes may indicate an increased risk of subsequent epilepsy and other adverse neurological outcomes [1,2,3]. Specifically, prolonged FS may be associated with hippocampal injury and later epileptogenesis in a subset of affected children [1,4]. Despite their high prevalence and clinical significance, the biological mechanisms underlying FSs, especially those related to phenotype heterogeneity and divergent prognosis, remain incompletely understood.

Current evidence suggests that the pathogenesis of FS is multifactorial, involving genetic susceptibility, impaired inhibitory neurotransmission, receptor- and ion channel-related dysfunction, and inflammatory signaling [4,5,6,7].

Studies have demonstrated that FS can facilitate epileptogenesis by disrupting GABAergic network maturation and hippocampal circuitry [5]. In addition, pathogenic variants have been identified in epilepsy-related genes, including GABRB3, highlighting the importance of inherited defects in inhibitory synaptic signaling in the FS spectrum [7]. It has also been shown that inflammatory mediators, specifically interleukin-1β, may contribute to lowering the seizure threshold and promoting FS generation [6,8]. However, these mechanisms do not fully explain why some children develop more severe seizure phenotypes or experience divergent long-term neurological outcomes. Therefore, identifying biologically meaningful pathways and phenotype-related biomarkers is essential for improving mechanistic understanding and risk stratification in FS.

The gut–brain axis is an important regulator of neurological health and disease. Through neural, endocrine, immune, and inflammatory pathways, gut microbiota can influence central nervous system function and neuronal excitability [9,10,11]. Gut-derived metabolites, such as neuroactive compounds and amino acid-related intermediates, can influence neurotransmission, blood–brain barrier integrity, and neuroinflammatory responses. Increasing evidence supports the involvement of gut microbiota in a range of neurological and neuropsychiatric disorders, including schizophrenia and autism spectrum disorder [12,13,14]. Furthermore, recent reports suggest that gut microbial communities undergo alterations in children with FS compared with healthy controls [15,16,17].

Metabolomics represents a powerful approach for identifying disease-associated biochemical alterations and candidate biomarkers. Of particular interest are amino acids, which serve not only as substrates for protein synthesis but also as precursors for neurotransmitters and immune-regulatory metabolites. Perturbations in amino acid metabolism—including disturbances in glutamine-, glutamate-, tryptophan-, kynurenine-, and GABA-related pathways—may directly affect neuronal excitability, disrupting the balance between excitatory and inhibitory neurotransmission [18,19,20,21,22,23,24]. In previous studies, children with FS were found to have abnormal levels of several amino acids and related metabolites, including phenylalanine, threonine, tryptophan, and lysine [15,25]. In addition, gut microbiota can modulate neuroactive compounds such as GABA, further supporting a mechanistic link between microbial dysbiosis and seizure susceptibility [22,23,24].

Although previous studies indicate that gut microbiota and metabolic alterations may play a role in FS development, most focus on comparisons between the overall FS population and healthy controls. It remains unclear whether simple FS (SFS) and complex FS (CFS) have a common gut-derived metabolic basis and whether phenotype-related metabolic divergence extends to the central nervous system. In the present study, we performed an integrated multiomics analysis combining gut microbiota profiling, serum amino acid analysis, fecal metabolomics, and cerebrospinal fluid (CSF) metabolomics. The aim was to identify gut–brain axis-related metabolic signatures associated with FS and to define phenotype-specific biological features with potential mechanistic and biomarker relevance.

## 2. Materials and Methods

### 2.1. Study Design

This study employed a case–control design to investigate the gut microbiota, serum amino acid profiles, fecal metabolome, and central (CSF) metabolome in children with febrile seizures (FSs). The main cohort comprised 100 children (50 FS cases and 50 age-/sex-matched healthy controls). FS cases were further subclassified into simple FS (SFS) and complex FS (CFS) cases based on standard clinical criteria. Fresh stool and venous blood samples were collected from all participants; for FS patients, blood was drawn within 24 h of the seizure episode. Multiomics profiling included (i) gut microbiota (16S rRNA sequencing) in the full 100-child cohort; (ii) serum amino acid profiling using a subset of samples (pooled into 25 FS vs. 25 control composites due to low volume constraints); (iii) untargeted fecal metabolomics in an expanded cohort (53 controls, 50 SFS cases, and 42 CFS cases); and (iv) untargeted CSF metabolomics comparing CFS and SFS. Each omics dataset was analyzed independently within its corresponding subset, and cross-omics integration was interpreted at the pathway and phenotype levels rather than as a fully paired individual-level analysis. Statistical methods included alpha/beta diversity, differential abundance, multivariate analysis (PCA/PLS-DA/OPLS-DA), and Spearman’s correlation.

### 2.2. Participants

Children aged between 6 months and 6 years were enrolled at the Children’s Hospital, Zhejiang University School of Medicine. The main cohort comprised 100 children: 50 children with FS and 50 healthy controls matched for age and sex. Following current pediatric clinical criteria, FSs were defined as fever-associated seizures in the absence of underlying neurological or systemic disorders. For phenotype-oriented analyses, FS cases were further classified into SFS or CFS cases according to standard clinical criteria. The following exclusion criteria were applied to minimize potential confounding effects on the microbiome and metabolome: (i) the use of antibiotics, probiotics, or prebiotics within 4 weeks prior to enrollment; (ii) a history of chronic gastrointestinal disorders (e.g., inflammatory bowel disease and chronic diarrhea); (iii) known metabolic, neurodegenerative, or epileptic disorders; and (iv) any systemic infection requiring intensive care. Dietary information was recorded for all participants but was not used as an exclusion criterion, as all children followed age-appropriate regular diets without therapeutic modifications. The study was approved by the Ethics Committee of the Children’s Hospital, Zhejiang University School of Medicine (2024-IRB-0089-P-01), on 2 April 2024. Written informed consent was obtained from the parents or legal guardians of all child participants.

### 2.3. Sample Collection and Allocation

Fresh stool samples were collected and stored at −80 °C until analysis. Venous blood samples were obtained during clinical visits. In the FS group, blood was collected within 24 h of the seizure episode to reflect the acute phase, whereas in the controls, blood was collected during routine health examinations. Approximately 3–5 mL of venous blood was drawn per child and processed to obtain serum, which was subsequently aliquoted and stored at −80 °C.

Lumbar puncture (LP) was performed in a subset of FS patients as part of standard clinical care to rule out central nervous system infections (e.g., meningitis or encephalitis). In all cases, LP was clinically indicated and not performed for research purposes alone. When sedation was required, it was administered according to institutional protocols using midazolam or chloral hydrate; no child received general anesthesia for the procedure. Residual cerebrospinal fluid (CSF) samples remaining after routine diagnostic testing were collected for research use, with written informed consent obtained from the parents or legal guardians for both the clinical procedure and the use of residual CSF for research purposes.

Gut microbiota profiling via 16S rRNA sequencing was performed in 50 children with FS and 50 healthy controls. Serum amino acid profiling was conducted using a subset of serum samples. Because some pediatric serum specimens did not meet the minimum input requirement of the analytical platform, an equal-volume pooling strategy was adopted to ensure analytical feasibility and reduce technical bias associated with ultra-low-volume samples. Specifically, two discrete serum samples from the same study group were combined into one composite sample, resulting in 25 pooled samples in the FS group and 25 pooled samples in the control group. To enhance the statistical power of phenotype-stratified comparisons, untargeted fecal metabolomics was performed in an expanded cohort. The final dataset included 53 healthy controls, 50 children with SFS, and 42 children with CFS. The central metabolic profiles of the CFS and SFS groups were compared using untargeted CSF metabolomics.

Owing to variations in sample size and composition across omics modules, each dataset was analyzed independently within its corresponding subset. Cross-omics integration was interpreted at the biological pathway and phenotype levels, rather than as a fully paired individual-level analysis.

### 2.4. 16S rRNA Gene Sequencing and Microbiota Analysis

Total DNA was extracted from stool samples using a QIAamp Fast DNA Stool Mini Kit (QIAGEN, Valencia, CA, USA). The Illumina platform (Illumina, Inc., San Diego, CA, USA) was employed to amplify and sequence the V4–V5 region of the bacterial 16S rRNA gene [26]. Sequencing data were processed using QIIME2 (version 2023.5), and sequences with 100% similarity were clustered into amplicon sequence variants (ASVs). Furthermore, representative sequences were classified using the Silva database. Alpha-diversity indices comprised the Richness, Shannon, Simpson, and Chao1 indices. Beta-diversity analysis, differential abundance testing, random forest, LEfSe, and KEGG-based functional prediction were conducted as previously described.

### 2.5. Serum Amino Acid Profiling

Serum amino acid and derivative/metabolite analysis was performed according to protocols established in the original study. In brief, 50 μL of serum was transferred into a 1.5 mL centrifuge tube, followed by the addition of 200 μL of methanol containing internal standards, dithiothreitol, and formic acid. After vortexing and centrifugation, the supernatant was obtained, and it was used for downstream metabolite analysis. Serum amino acid profiling was performed via ion-exchange chromatography using an automatic amino acid analyzer (Hitachi L-8900, Hitachi High-Technologies Corporation, Tokyo, Japan) equipped with a cation-exchange column (4.6 × 60 mm, Hitachi High-Technologies Corporation, Tokyo, Japan), with a stepwise gradient of lithium citrate buffers at a flow rate of 0.4 mL/min and post-column ninhydrin derivatization for detection at 570 nm and 440 nm. Data acquisition and peak integration were performed using the instrument’s dedicated software (version 1.0). Norleucine was used as an internal standard for quantification, and concentrations were calculated based on standard calibration curves for each amino acid. Serum amino acid profiling was performed via ion-exchange chromatography [27]. Global metabolic separation was evaluated using PCA and PLS-DA, and differential metabolites were identified based on statistical comparisons between groups.

### 2.6. Untargeted Fecal Metabolomics

The expanded cohort, including healthy controls, patients with SFS, and patients with CFS, was subjected to untargeted fecal metabolomic profiling by PANOMIX Biomedical Tech Co., Ltd. (Suzhou, China). Briefly, fecal samples were thawed on ice, and metabolites were extracted using methanol/water (4:1, *v*/*v*) containing internal standards. After vortex mixing, sonication, and centrifugation at 4 °C, the supernatant was collected, dried under nitrogen gas, and reconstituted in 100 µL of 50% methanol for LC–MS/MS analysis.

Chromatographic separation was performed on an ACQUITY UPLC system equipped with an ACQUITY UPLC HSS T3 column (2.1 × 100 mm, 1.8 µm; Waters, Milford, MA, USA). The column temperature was maintained at 40 °C. The mobile phases consisted of solvent A, 0.1% formic acid in water, and solvent B, 0.1% formic acid in acetonitrile. Gradient elution was performed at a flow rate of 0.3 mL/min according to the optimized analytical protocol of the service provider. Mass spectrometry was performed using a Q Exactive Orbitrap mass spectrometer (Thermo Fisher Scientific, Waltham, MA, USA) equipped with an electrospray ionization source. Samples were analyzed in both positive- and negative-ion modes. Full-scan MS data were acquired over an *m*/*z* range of 70–1000 at a resolution of 70,000 at *m*/*z* 200.

To ensure analytical stability and reproducibility, pooled quality control (QC) samples were prepared by mixing equal aliquots from all study samples, and they were injected into every 10 samples throughout the analytical run. Blank samples were included to monitor background signals, and the sample injection order was randomized to reduce batch-related bias. Matrix effects and instrumental variation were corrected using internal-standard normalization. Metabolite features with a coefficient of variation greater than 30% in the QC samples were excluded from downstream analysis.

Raw LC–MS data were processed using the metabolomics data processing workflow of the BioDeep platform, including peak detection, retention time alignment, peak integration, normalization, and feature filtering. Normalized peak intensities were log2-transformed before statistical analysis. Metabolite annotation was performed by matching accurate mass, retention time, isotope distribution, and MS/MS fragmentation information against the service provider’s in-house library and public metabolite databases, including KEGG, HMDB, METLIN, and MassBank, where applicable. Principal component analysis (PCA) and orthogonal partial least-squares discriminant analysis (OPLS-DA) were used to evaluate global metabolic separation among groups. Differential metabolites were identified based on fold change, multivariate model contribution, and statistical significance after Benjamini–Hochberg false discovery rate correction. KEGG pathway enrichment analysis was performed using annotated differential metabolites.

### 2.7. Untargeted CSF Metabolomics

The central metabolic profiles of the CFS and SFS groups were compared using untargeted CSF metabolomics. CSF samples (100 µL) were processed using the same extraction procedure as described for fecal metabolomics. Chromatographic and mass spectrometric conditions were identical to those used for fecal metabolomics, employing the same column, mobile phases, gradient program, and MS parameters. The same quality control procedures as described for fecal metabolomics were applied, including regular injection of pooled QC samples, internal standard normalization for matrix effect correction, and exclusion of features with a QC coefficient of variation > 30%. PCA and OPLS-DA were used to examine global metabolic separation, and differential CSF metabolites between the two FS phenotypes were identified using volcano plots combined with clustering analyses.

### 2.8. Correlation Analysis

Relationships between differential microbial taxa and serum metabolites were assessed using Spearman’s correlation analysis. Correlation heatmaps and network representations were used to identify key taxa–metabolite interactions.

### 2.9. Statistical Analysis

Continuous variables were compared using Student’s *t*-test or the Mann–Whitney *U* test, as appropriate. Multiple-group comparisons were performed using a one-way analysis of variance (ANOVA) or the Kruskal–Wallis test with post hoc correction. Beta-diversity differences were evaluated using PERMANOVA. Given the differences in sample size across omics platforms, each omics dataset was analyzed independently within its corresponding eligible subset. Cross-omics integration was interpreted primarily at the biological pathway and phenotype stratification levels, rather than as a fully paired, individual-level multiomics comparison.

For differential analyses involving untargeted metabolomics (fecal and CSF) and microbial differential abundance testing, multiple-testing correction was performed using the Benjamini–Hochberg false discovery rate (FDR) method, with adjusted q-values re-ported alongside raw *p*-values where available. For serum amino acid profiling, which involved a smaller targeted panel of metabolites, Bonferroni correction was applied to control the family-wise error rate. Differential metabolites and microbial taxa were interpreted as statistically significant when the adjusted q-value was <0.05 (or Bonferroni-corrected *p* < 0.05 for serum amino acids), unless otherwise specified. Raw *p*-values displayed in figures are nominal values and should be interpreted together with the corresponding multiple-testing correction strategy.

Regarding confounder adjustment, age and sex were matched between the FS and control groups at enrollment. For the expanded fecal metabolomics cohort, we further adjusted for age and sex using linear models where applicable to minimize residual confounding. No adjustment was made for diet or antibiotic/probiotic use, as these factors were explicitly addressed through the exclusion criteria described in the Participants Subsection. Given the differences in sample size across omics platforms, each omics dataset was analyzed independently within its corresponding eligible subset. Cross-omics integration was interpreted primarily at the biological pathway and phenotype stratification levels, rather than as a fully paired, individual-level multiomics comparison. All statistical analyses were performed using R software (version 4.2.0) and GraphPad Prism 9.0.

## 3. Results

### 3.1. Study Population and Multiomics Workflow

The main cohort included 50 children with FS and 50 healthy controls, and the gut microbiota was profiled in these two groups. Owing to limited serum availability in a subset of pediatric samples, serum amino acid analysis was performed by pooling two samples from the same study group to generate a single composite specimen, resulting in 25 pooled FS samples and 25 pooled control samples. For phenotype-stratified fecal metabolomics, an expanded cohort was established, including 53 healthy controls, 50 patients with SFS, and 42 patients with CFS. In addition, the central metabolic profiles of the two FS phenotypes, comprising 20 patients with SFS and 22 patients with CFS, were compared using untargeted CSF metabolomics. The present study represents a stratified multiomics investigation based on eligible subsets for each platform, rather than a fully matched dataset across all omics layers.

### 3.2. Microbiota Diversity and Composition

#### 3.2.1. Alpha and Beta Diversity

**Figure 1:**
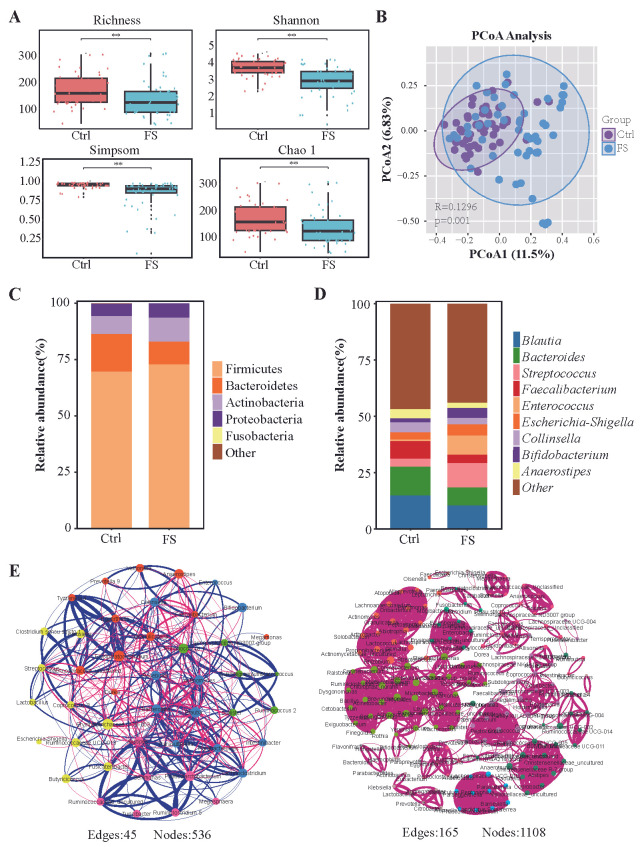
Microbiota diversity and composition in patients with FS. (**A**) α-Diversity analysis: Richness, Shannon, Simpson, and Chao1. (**B**) β-Diversity analysis using principal coordinate analysis (PCoA) based on Bray–Curtis distances. (**C**) Phylum level. (**D**) Genus level. (**E**) Correlation network of gut microbiota at the genus level in the control and FS groups. Red lines indicate positive correlations, and blue lines indicate negative correlations. The size of each node represents the number of significant associations with other taxa (larger node = more connections), ** *p *< 0.01.

To investigate whether FSs induce intestinal dysbiosis, the composition of the gut microbiota was evaluated. The α-diversity indices—including Richness, Shannon, Simpson, and Chao1 indices—were significantly lower in the FS group than in the control group (*p* < 0.01) (Figure 1A), suggesting a less diverse and more imbalanced microbial community. Moreover, PCoA based on Bray–Curtis distances revealed distinct clustering between the FS and control groups (R = 0.1296, *p* = 0.01) (Figure 1B), indicating a significant alteration in the microbial community structure.

#### 3.2.2. Phylum and Genus Levels

At the phylum level, the gut microbiota in both groups was dominated by *Firmicutes*, *Bacteroidetes*, *Actinobacteria*, and *Proteobacteria*. The relative abundances of *Firmicutes* and *Actinobacteria* were significantly increased in the FS group, whereas *Bacteroidetes* were more abundant in the control group (Figure 1C).

At the genus level, further analysis revealed that the dominant genera in the control group were *Blautia*, *Bacteroides*, *Faecalibacterium*, and *Collinsella*, while those in the FS group were *Streptococcus*, *Blautia*, *Bacteroides*, *Enterococcus*, and *Escherichia–Shigella* (Figure 1D). Notably, in the FS group, the relative abundance of *Streptococcus* was significantly higher, whereas that of *Blautia*, *Bacteroides*, and *Faecalibacterium* was lower.

#### 3.2.3. Correlation Network of Gut Microbiota

A microbial interaction network was constructed based on the correlation analysis of the gut microbiota in the control and FS groups. Significant correlations were defined as those with an absolute correlation coefficient greater than 0.5 and a *p* value lower than 0.05. The FS group exhibited a more complex interaction network than the control group, as reflected by a higher modularity class value (0.633 vs. 0.14) (Figure 1E), indicating more intricate microbial interactions and greater interaction diversity.

#### 3.2.4. Differential Analysis of Gut Microbiota

**Figure 2:**
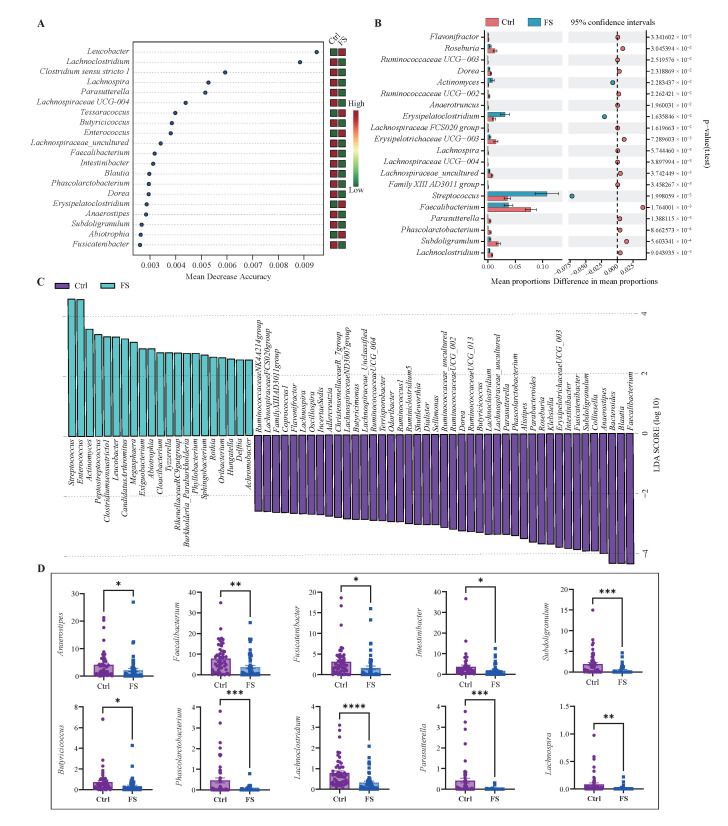
Differential analysis between the FS and control groups. (**A**) Random Forest analysis. (**B**) Statistical validation. (**C**) LEfSe differential analysis (LDA score). (**D**) Statistical comparison between the groups (box plots), * *p* < 0.05, ** *p* < 0.01, *** *p* < 0.001, and **** *p* < 0.0001.

Differential microbial biomarkers were identified through a rigorous multi-step analytical pipeline to ensure robustness and biological relevance. First, a Random Forest classifier was employed to rank microbial features based on their importance scores, prioritizing taxa with the highest discriminatory power between the FS and control groups. The analysis revealed significant differences in MDA values among the genera, reflecting their varying predictive contributions. *Leucobacter* exhibited the highest MDA value at approximately 0.009, followed by *Lachnoclostridium*, *Clostridium sensu stricto 1*, *Lachnospira*, and *Parasutterella*. Notably, *Leucobacter*, *Tessaracoccus*, *Enterococcus*, and *Erysipelatoclostridium* were more abundant in the FS group, whereas *Lachnoclostridium*, *Clostridium sensu stricto 1*, *Lachnospira*, and *Parasutterella* predominated in the control group (Figure 2A).

Next, statistical validation was performed using differential abundance analysis to confirm significant differences (*p* < 0.05) in microbial abundances. The analysis revealed variations in microbial abundance between the control and FS groups at both the phylum and genus levels. Notably, the FS group exhibited a significantly higher mean proportion of *Streptococcus*, *Erysipelatoclostridium*, and *Actinomyces* (*p* < 0.01) and a significantly lower mean proportion of *Faecalibacterium*, *Lachnoclostridium*, *Subdoligranulum*, and *Parasutterella* than the control group (*p* < 0.01) (Figure 2B). The 95% confidence intervals presented alongside the mean differences underscore the precision of these estimates.

Subsequently, LEfSe analysis (LDA score > 3.0, *p* < 0.01) was conducted to identify group-specific microbial signatures, ensuring biological interpretability. In the control group, *Faecalibacterium*, *Blautia*, *Bacteroides*, *Lachnoclostridium*, and *Parasutterella* were significantly enriched. In contrast, the FS group exhibited notably higher abundances of *Streptococcus*, *Enterococcus*, *Actinomyces*, *Peptostreptococcus*, and *Clostridium sensu stricto 1* (Figure 2C).

Only microbes consistently significant across all three methods were retained as candidate biomarkers, while low-abundance or method-specific hits were excluded to minimize false positives. Finally, box plots were used to visually and statistically validate the selected taxa, reinforcing their potential as reliable biomarkers for further investigation. We retained 10 microorganisms as candidate biomarkers, including *Faecalibacterium*, *Subdoligranulum*, *Lachnoclostridium*, *Parasutterella*, and *Phascolarctobacterium* (Figure 2D). These findings underscore the potential of these microbial species as biomarkers for distinguishing between the control and FS groups.

### 3.3. Gut Microbiota Function Analysis Based on KEGG

**Figure 3:**
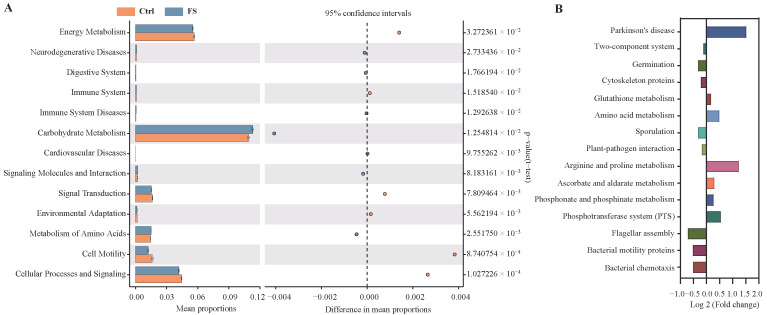
Differential analysis of biological processes and pathways between the control and FS groups using KEGG analysis. (**A**) Comparison of mean proportions of biological processes. Data are presented as mean proportions with 95% confidence intervals, and significant differences are indicated by *p* values from *t* tests. (**B**) Fold change in KEGG pathways. Positive values indicate upregulation in the FS group, and negative values indicate downregulation.

KEGG functional prediction was applied to elucidate the metabolic pathways and functions potentially involved in the alterations of the gut microbiome in FS. The mean proportions and their differences across various functional categories—including energy metabolism, neurodegenerative diseases, digestive system, immune system diseases, and carbohydrate metabolism—are presented in Figure 3A. Significant differences between the groups were observed in cellular processes and signaling, cell motility, and amino acid metabolism. An analysis of specific metabolic pathways, represented as log2 fold changes, predicted alterations in arginine and proline metabolism, amino acid metabolism, and glutathione metabolism, suggesting that FS is associated with alterations in these pathways (Figure 3B). It is important to note that these KEGG predictions are derived from 16S rRNA sequencing data using PICRUSt-like algorithms and are therefore indirect; they should be interpreted as predicted functional alterations rather than direct measurements of metabolic activity, and orthogonal validation via metatranscriptomics or targeted metabolomics is warranted.

### 3.4. FS Showed Systemic Amino Acid Metabolic Remodeling

**Figure 4:**
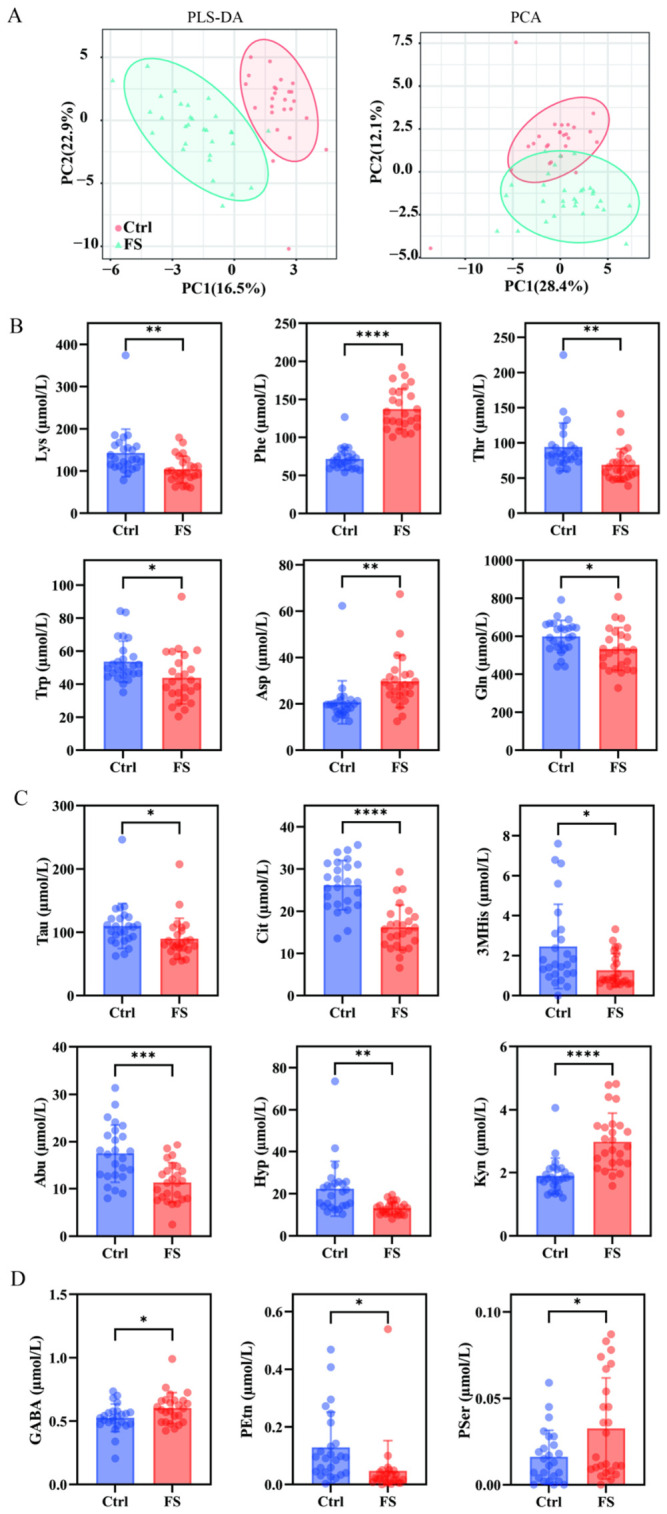
Serum analysis of free amino acids and their derivatives and metabolites. (**A**) PLS-DA Score and Principal component analysis (PCA) score. (**B**) Differential free amino acid levels in the serum of the control and FS groups. (**C**,**D**) Differential levels of free amino acid derivatives in the serum of the control and FS groups, * *p* < 0.05, ** *p* < 0.01, *** *p* < 0.001, and **** *p* < 0.0001.

Based on the KEGG results, we further analyzed serum free amino acids and their derivatives and metabolites. The PLS-DA model comparing FS and control samples contained three components and showed R2X(cum) = 0.45, R2Y(cum) = 0.926, and Q2(cum) = 0.8, indicating good model fit and predictive performance (Supplementary Table S1). The PCA plot demonstrates a clear separation between the control and FS groups along both principal components, with the control group positioned on the left and the FS group positioned on the right (Figure 4A), indicating significant differences between the groups.

An analysis of the serum free amino acid profile revealed that, among the essential amino acids, the FS group exhibited decreased serum levels of threonine, tryptophan, and lysine (*p* < 0.05) (Figure 4B) and increased serum levels of phenylalanine (*p* < 0.05) (Figure 4B). In addition, among the nonessential amino acids, the FS group showed a significant decrease in serum glutamine (*p* < 0.05) and an increase in aspartic acid (*p* < 0.05) (Figure 4B).

In terms of amino acid derivatives and metabolites, the FS group exhibited significantly reduced levels of taurine, citrulline, 3-methylhistidine, α-aminobutyric acid, hydroxyproline, and PEtn (all *p* < 0.05; Figure 4C) and significantly elevated levels of kynurenine, GABA, and phosphoserine compared to the control group (Figure 4D).

### 3.5. Candidate Neuroactive Signatures Identified by Microbe–Metabolite Associations

**Figure 5:**
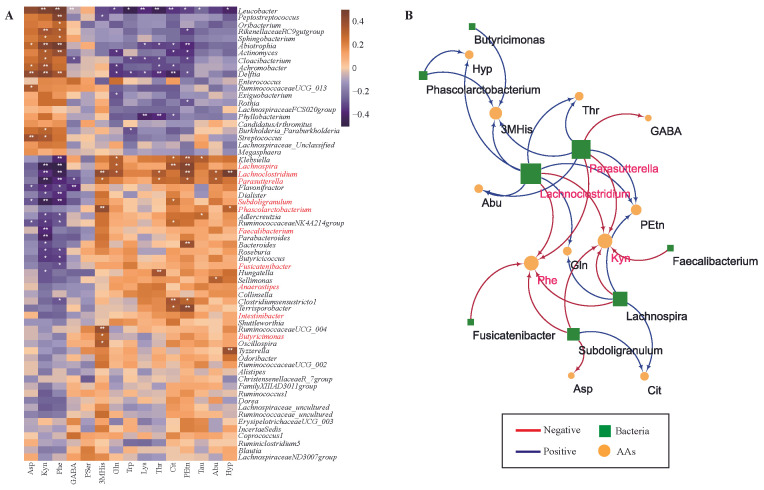
Correlation heat map of serum metabolites and gut microbiota. (**A**) Each cell represents a correlation coefficient, with the color scale ranging from blue (negative correlation) to pink (positive correlation). Asterisks indicate the level of statistical significance: * *p* < 0.05 and ** *p* < 0.01. (**B**) Network of interactions between bacteria (represented by squares), amino acids (represented by circles), and the positive (blue)/negative (red) interactions between them. The size of the squares (representing bacteria) and circles (representing amino acids) likely indicates the significance or impact of each entity in the network.

Based on previous research, we further examined the correlations between differential amino acids and their derivatives and the gut microbiota. Our analysis revealed significant associations between specific amino acids and microbial taxa, with distinct interaction patterns (Figure 5A). Notably, phenylalanine and kynurenine exhibited the strongest associations, predominantly showing negative correlations (Figure 5B), which suggests their potential involvement in microbial metabolism. At the microbial level, *Lachnoclostridium* and *Parasutterella* emerged as key taxa associated with the amino acid profiles. In particular, *Parasutterella* was negatively correlated with GABA, underscoring its potential role in modulating or responding to amino acid metabolism within the gut environment.

### 3.6. Phenotype-Related Metabolic Remodeling in SFS and CFS Revealed by Fecal Metabolomics

Having established that children with FS exhibit significant gut microbial dysbiosis and systemic amino acid metabolic disturbances compared with healthy controls, we next sought to determine whether the two clinically distinct FS subtypes—simple FS (SFS) and complex FS (CFS), which differ in prognosis and risk of long-term neurological sequelae—possess distinguishable gut-derived metabolic signatures. To this end, untargeted fecal metabolomics was performed in an expanded cohort comprising 53 healthy controls, 50 children with SFS, and 42 children with CFS. Pairwise comparisons were conducted among the three groups to delineate phenotype-specific metabolic alterations.

**Figure 6:**
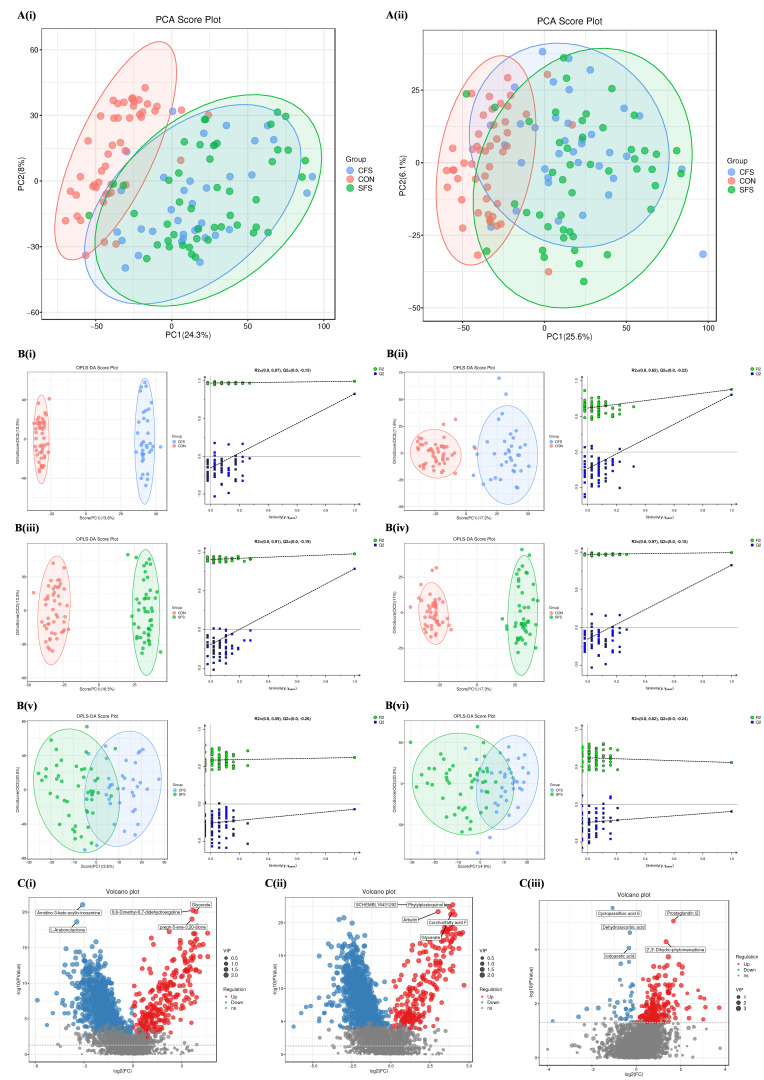
PCA and OPLS-DA score plots of fecal metabolites among different groups. (**A**(**i**)) PCA score plot (positive). (**A**(**ii**)) PCA score plot (negative). (**B**(**i**,**iii**,**v**)) OPLS-DA score plot (positive). (**B**(**ii**,**iv**,**vi**)) OPLS-DA score plot (negative). (**C**) Volcano plots of fecal differential metabolites. (**i**) CFS vs. CON. (**ii**) SFS vs. CON. (**iii**) CFS vs. SFS.

Principal component analysis (PCA) showed distinct group separation among the control, SFS, and CFS groups (Figure 6A). Notably, orthogonal partial least-squares discriminant analysis (OPLS-DA) clearly separated all three groups (Supplementary Table S2), and, importantly, CFS could be distinguished from SFS, indicating distinct molecular profiles between simple and complex febrile seizures (Figure 6B). Volcano plots demonstrated substantial numbers of differential metabolites in both CFS versus control and SFS versus control comparisons (Figure 6C). Collectively, these results indicate that both SFS and CFS deviate from normal gut-derived metabolic homeostasis while also exhibiting phenotype-specific metabolic features.

### 3.7. Arginine Biosynthesis as a Shared Altered Pathway Across FS Phenotypes

**Figure 7:**
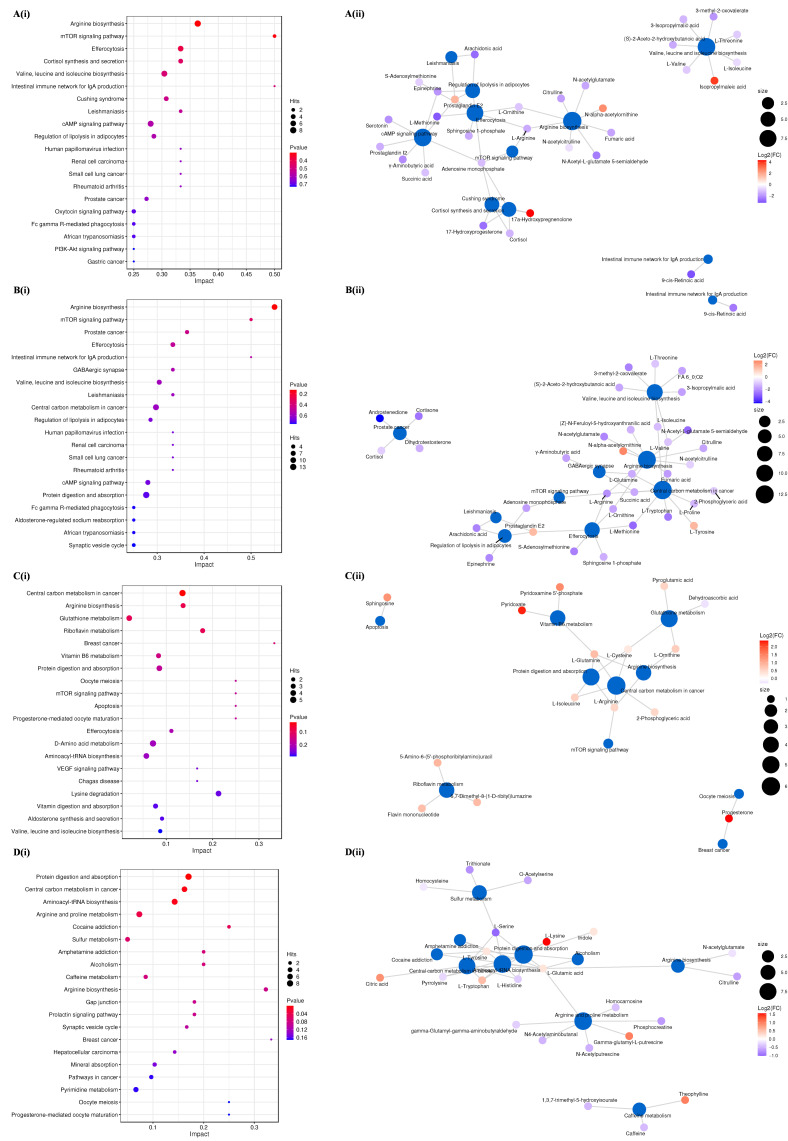
KEGG enrichment analysis of differential metabolites. (**A**(**i**,**ii**)) Fecal enrichment bubble plot and Network diagram (CFS vs. CON). (**B**(**i**,**ii**)) SFS vs. CON. (**C**(**i**,**ii**)) CFS vs SFS. (**D**(**i**,**ii**)) CSF enrichment bubble plot and Network diagram (CFS vs. SFS).

KEGG enrichment analysis revealed that arginine biosynthesis was a significantly altered pathway in both CFS versus control and SFS versus control comparisons, with L-arginine levels significantly lower in the patient groups than in the healthy controls (Figure 7A–C). This convergence suggests that arginine metabolism may serve as a core shared metabolic backbone in FS, linking gut dysbiosis and metabolic vulnerability across clinical phenotypes.

### 3.8. Central Metabolic Signatures Distinguishing CFS from SFS Identified via CSF Metabolomics

**Figure 8:**
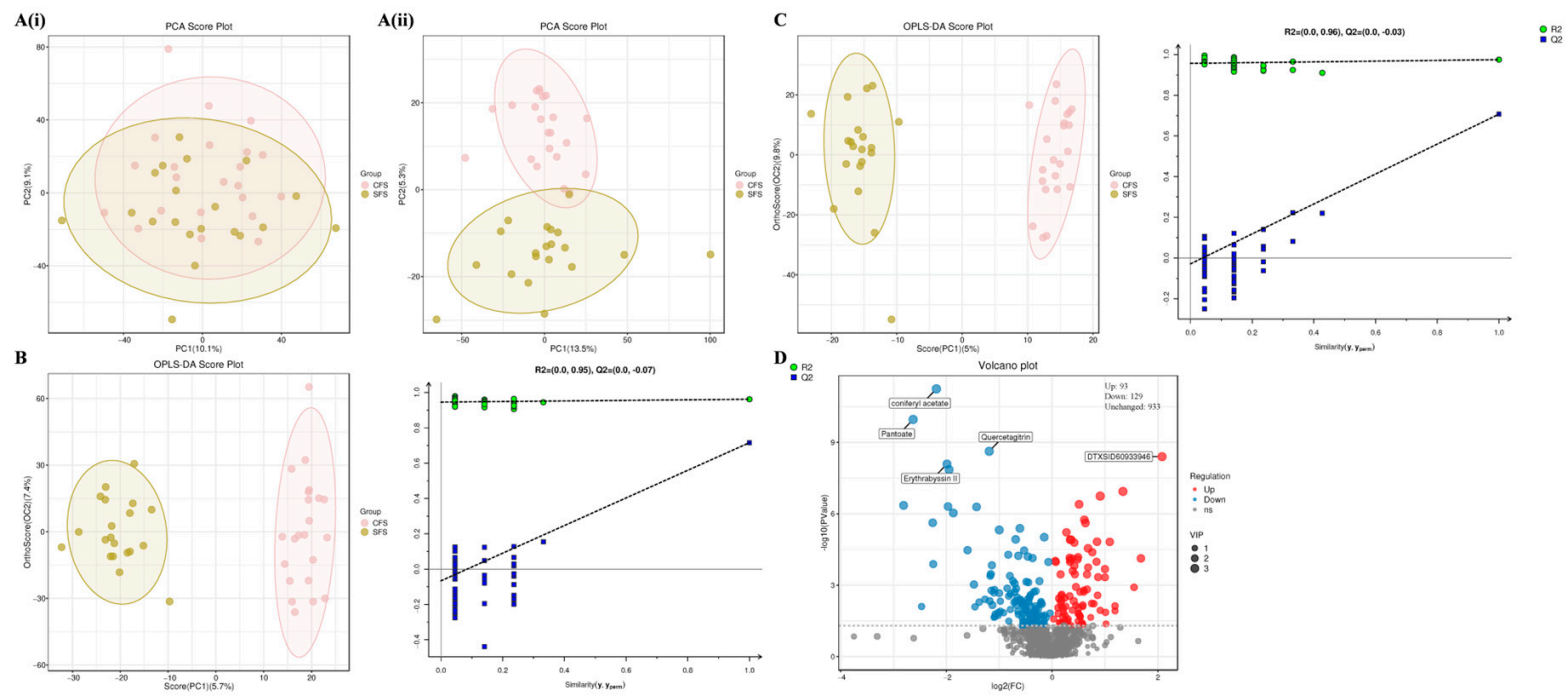
Differential metabolites in CSF between the CFS and SFS groups. (**A**(**i**)) PCA score plot (positive). (**A**(**ii**)) PCA score plot (negative). (**B**) OPLS-DA score plot and permutation test (positive). (**C**) OPLS-DA score plot and permutation test (negative). (**D**) Volcano plots.

To determine whether phenotype-specific peripheral changes extend to the central nervous system, CFS and SFS were compared using CSF metabolomics. PCA and OPLS-DA demonstrated distinct clustering between the two groups (Supplementary Table S3), and volcano plots combined with clustering analysis further supported the presence of differential CSF metabolites (Figure 8A–C). These data indicate that the biological differences between SFS and CFS also involve the central metabolic microenvironment rather than being confined to the gut and peripheral circulation. KEGG analysis predicted significant alterations in arginine metabolism, suggesting that this pathway may play a crucial role in the underlying mechanisms of FS (Figure 7D). A full list of differential CSF metabolites between CFS and SFS, along with their fold changes, raw *p*-values, FDR-adjusted q-values, and KEGG pathway annotations, is provided in Supplementary Table S4.

### 3.9. Gut–Brain Axis Dysregulation in FS Supported by Integrated Multiomics

Overall, the findings support a layered model of FS pathophysiology. At the shared disease level, FS is characterized by gut microbial dysbiosis and systemic amino acid disturbances. At the phenotype level, both SFS and CFS exhibit altered fecal metabolomes with involvement of arginine biosynthesis. At the central level, CSF metabolomics distinguishes CFS from SFS, indicating that phenotype heterogeneity reflects varying degrees of propagation of peripheral abnormalities into the central nervous system.

## 4. Discussion

In this study, we used an integrated multiomics strategy to investigate gut microbiota alterations, systemic amino acid remodeling, and phenotype-related metabolic signatures in children with FS. From our data, three main conclusions were drawn: First, FS was associated with gut microbial dysbiosis accompanied by broad disturbances in amino acid-related metabolism. Second, both SFS and CFS showed a disrupted gut metabolic background, with arginine biosynthesis emerging as a shared altered pathway. Third, CSF metabolomics revealed central metabolic divergence between SFS and CFS, signifying that clinical phenotype heterogeneity in FS may reflect different depths of biological disturbance along the gut–brain axis.

Our microbiota data support the view that FS is associated with intestinal dysbiosis. The enrichment of *Streptococcus*, *Enterococcus*, and related taxa, together with the depletion of *Faecalibacterium*, *Lachnoclostridium*, and *Parasutterella*, indicates a shift from a relatively homeostatic microbial ecosystem toward a potentially pro-inflammatory and metabolically perturbed state [28,29]. This interpretation aligns with that of previous studies indicating that FS is accompanied by gut microbiota alterations in both children and experimental models [15,17]. More broadly, accumulating evidence suggests that gut microbial communities can influence brain function through immune, endocrine, metabolic, and neural signaling pathways within the gut–brain axis [30,31,32]. In this context, the central position of *Lachnoclostridium* and *Parasutterella* in our taxo-metabolite correlation network is noteworthy, as these may actively participate in host metabolic regulation rather than merely reflecting passive compositional changes [23,33,34,35,36,37].

In this context, our KEGG-based functional prediction based on 16S data suggested that FS is associated with alterations in amino acid-related metabolic pathways, particularly those for arginine and proline. However, it should be emphasized that these predictions are derived indirectly from taxonomic profiles using PICRUSt-like algorithms and do not constitute direct evidence of altered metabolic activity. We therefore interpret these findings as hypothesis-generating observations that point toward pathways worthy of targeted investigation using complementary approaches such as metatranscriptomics or stable-isotope tracing studies. With this caveat in mind, we next turned to direct serum metabolite measurements to assess amino acid remodeling. The serum metabolite profile suggests that FS is accompanied by marked amino acid metabolic remodeling. Elevated phenylalanine and kynurenine, reduced tryptophan and glutamine, and altered GABA-related balance collectively indicate disturbances in inflammatory and neuroactive pathways [25,38,39,40]. The reduction in multiple amino acid derivatives further implies broader metabolic instability during the acute phase of FS. These observations are consistent with a mechanism wherein interactions between gut dysbiosis and host amino acid metabolism influence neuronal excitability and seizure susceptibility [15,41].

Furthermore, our phenotype-stratified analysis lends additional support to the biological relevance of the observed microbial and metabolic disturbances. Although both SFS and CFS showed common alterations in fecal metabolomes compared with controls, they also exhibited significant metabolic separation from each other. This divergence indicates that gut-derived metabolic changes are not uniform across FS subtypes but instead align with clinically defined phenotype severity [15,17]. Given that the gut microbiota is a major determinant of fecal metabolic composition [28,29], the distinct metabolic profiles between SFS and CFS indirectly reinforce the notion that the intestinal ecosystem is differentially affected in these two clinical groups. Thus, while we cannot definitively attribute causality, the phenotype-dependent metabolic stratification observed in our data argues against the microbial and metabolic shifts being merely nonspecific byproducts of fever; rather, they appear to track with meaningful clinical heterogeneity, further supporting their potential relevance to FS pathophysiology.

An important conceptual contribution of the present work is the extension of a conventional two-group comparison to phenotype-oriented analysis. Although both SFS and CFS showed marked fecal metabolic divergence from controls, arginine biosynthesis frequently emerged as a shared altered pathway in both comparisons, and L-arginine was reduced in both phenotypes. This convergence indicates that arginine metabolism may represent a common metabolic backbone in FS rather than a change restricted to one clinical subtype. As arginine metabolism is closely associated with nitric oxide production, immune signaling, vascular regulation, and neuronal homeostasis, disruption of this pathway may constitute a biologically relevant hub connecting gut-derived metabolic perturbation to seizure vulnerability. Hence, arginine metabolism should be considered a candidate mechanistic pathway of translational interest in FS and warrants further targeted validation [42,43].

Notably, CSF metabolomics showed that SFS and CFS also differed at the central level. This insight extends the phenotype-specific signal beyond the gut and peripheral circulation, supporting the possibility that more severe FS phenotypes are associated with a deeper degree of central biochemical disturbance [44,45]. Within this framework, SFS and CFS may have a common gut-derived and peripheral metabolic basis, while CFS may represent a state in which these abnormalities propagate more substantially into the central compartment. This interpretation aligns with prior evidence that prolonged or severe FS phenotypes, particularly febrile status epilepticus within the complex FS spectrum, are more strongly associated with subsequent adverse neurological outcomes [46,47].

From a translational perspective, the combined microbial and metabolic alterations identified in this study may provide candidate biomarkers for phenotype stratification and risk assessment in pediatric FS [48]. The integration of gut microbiota profiling with serum, fecal, and CSF metabolic signatures may improve mechanistic interpretation beyond single-omics approaches, thereby helping guide future studies aimed at the early identification of high-risk phenotypes [48,49].

Several limitations of this study should be acknowledged. First, sample sizes varied across omics modules due to specimen availability, and serum amino acid profiling was performed on pooled samples rather than individual sera because many pediatric specimens failed to meet the minimum volume requirement. While this pooling strategy reduced technical bias and allowed the inclusion of otherwise excluded samples, it inherently precludes individual-level correlation analyses and limits sensitivity to inter-individual heterogeneity; therefore, serum amino acid findings should be interpreted as group-level trends. Second, as a consequence, our cross-omics integration should be interpreted primarily at the biological pathway and phenotype levels rather than as fully paired individual-level analyses. Third, the potential influence of medication and fever should be considered. All FS patients received standard antipyretics (acetaminophen or ibuprofen) but no anticonvulsants prior to sampling. Although short-term antipyretic use is not known to markedly alter gut microbiota or systemic metabolites, the acute inflammatory state associated with fever itself may have contributed to the observed changes, and the effects of fever, seizure, and medication cannot be fully disentangled in this acute-presentation design. Finally, the specific CSF metabolites that best distinguish CFS from SFS require further targeted validation and annotation.

## 5. Conclusions

Despite these limitations, the present study provides a coherent framework linking gut dysbiosis, amino acid remodeling, fecal metabolic stratification, and central metabolic divergence in FS. Children with FS exhibit gut microbial dysbiosis, systemic amino acid disturbances, and phenotype-related metabolic stratification. Arginine biosynthesis may represent a shared altered pathway across FS phenotypes, while CSF metabolomics shows additional central metabolic signatures that differentiate CFS from SFS. This integrated model helps elucidate the differing clinical outcomes of SFS and CFS and provides a rationale for future biomarker discovery and mechanism-based therapeutic exploration [50].

## Data Availability

The data supporting the findings of this study are available from the corresponding author upon reasonable request. All sequence data are available from the NCBI Sequence Read Archive under BioProject ID: PRJNA1245040.

## Supplementary Materials

The following supporting information can be downloaded at: https://www.mdpi.com/article/10.3390/biomedicines14071568/s1, Table S1: OPLS-DA model comparing serum amino acid profiles between FS and control groups; Table S2: OPLS-DA model comparing fecal untargeted metabolomic profiles among CFS, SFS, and control groups; Table S3: OPLS-DA model comparing CSF untargeted metabolomic profiles between CFS and SFS groups; Table S4: The full list of differential CSF metabolites between CFS and SFS.

## Abbreviations

FS	febrile seizures
SFS	simple febrile seizures
CFS	complex febrile seizures
CSF	cerebrospinal fluid
GABA	γ-aminobutyric acid
PCA	principal component analysis
PLS-DA	partial least squares discriminant analysis
OPLS-DA	orthogonal partial least squares discriminant analysis
KEGG	Kyoto Encyclopedia of Genes and Genomes
LEfSe	linear discriminant analysis effect size
ASV	amplicon sequence variant
